# SARS-CoV-2 spike-protein D614G mutation increases virion spike density and infectivity

**DOI:** 10.1038/s41467-020-19808-4

**Published:** 2020-11-26

**Authors:** Lizhou Zhang, Cody B. Jackson, Huihui Mou, Amrita Ojha, Haiyong Peng, Brian D. Quinlan, Erumbi S. Rangarajan, Andi Pan, Abigail Vanderheiden, Mehul S. Suthar, Wenhui Li, Tina Izard, Christoph Rader, Michael Farzan, Hyeryun Choe

**Affiliations:** 1 Department of Immunology and Microbiology, The Scripps Research Institute, Jupiter, FL USA; 2 Department of Integrative Structural and Computational Biology, The Scripps Research Institute, Jupiter, FL USA; 3 Department of Pediatrics, Division of Infectious Disease, Emory University School of Medicine, Atlanta, GA USA; 4 Emory Vaccine Center, Emory University School of Medicine, Atlanta, GA USA; 5 Yerkes National Primate Research Center, Atlanta, GA USA; 6 Emory-UGA Center of Excellence of Influenza Research and Surveillance (CEIRS), Atlanta, GA USA; 7 National Institute of Biological Sciences, Tsinghua Institute of Multidisciplinary Biomedical Research, Tsinghua University, Beijing, China

**Keywords:** Virology, SARS-CoV-2

## Abstract

SARS-CoV-2 variants with spike (S)-protein D614G mutations now predominate globally. We therefore compare the properties of the mutated S protein (S^G614^) with the original (S^D614^). We report here pseudoviruses carrying S^G614^ enter ACE2-expressing cells more efficiently than those with S^D614^. This increased entry correlates with less S1-domain shedding and higher S-protein incorporation into the virion. Similar results are obtained with virus-like particles produced with SARS-CoV-2 M, N, E, and S proteins. However, D614G does not alter S-protein binding to ACE2 or neutralization sensitivity of pseudoviruses. Thus, D614G may increase infectivity by assembling more functional S protein into the virion.

## Abstract (web summary)

SARS-CoV-2 variants with spike (S)-protein D614G mutations currently predominate globally. Here, Zhang et al. hypothesize that D614G variant may increase infectivity by increasing S protein abundance on the virion since pseudoviruses carrying S-G614 incorporate higher amounts of S protein and enter cells more efficiently than those carrying S-D614.

## Introduction

Until late 2019, only six coronaviruses were known to infect humans: HCoV-229E, HCoV-OC43, SARS-CoV (SARS-CoV-1), HCoV-NL63, CoV-HKU1, and MERS-CoV. A seventh human coronavirus, i.e., SARS-CoV-2 detected in late 2019 is closely related to SARS-CoV-1 that emerged in the winter of 2002/2003.

The coronavirus spike (S) protein mediates receptor binding and fusion of the viral and cellular membrane. The S protein extends from the viral membrane and is uniformly distributed as trimers on the virion surface to give the appearance of a crown (corona in Latin). The coronavirus S protein is divided into two domains: S1 and S2. The S1 domain mediates receptor binding and the S2 mediates downstream membrane fusion^1,2^. The receptor for SARS-CoV-2 is angiotensin-converting enzyme 2 (ACE2)^3–7^, a metalloprotease that also serves as the receptor for SARS-CoV-1^8^. A small, independently folded subdomain of S1, described as the receptor-binding domain (RBD), directly binds ACE2 when the virus engages a target cell^9–12^. The S1–S2 junction of SARS-CoV-2 is processed by a furin-like proprotein convertase in the virus-producing cell. In contrast, the S1–S2 junction of SARS-CoV-1 is processed by TMPRSS2 at the cell surface or by lysosomal cathepsins in the target cells^13–18^. The S proteins of both viruses are further processed in the target cell within the S2 domain at the S2ʹ site, an event that is also required for productive infection^19,20^.

**Fig. 1:**
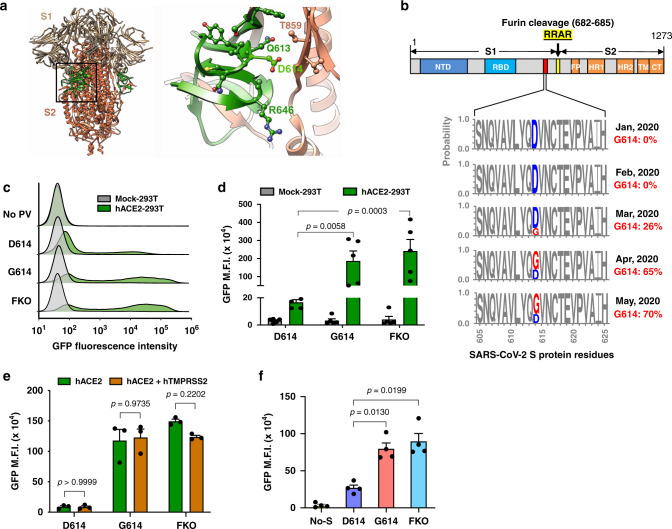
The D614G mutation is associated with enhanced infectivity. **a** Cryo-EM structure of S1 (gray) and S2 (orange) heterodimer (PBD entry 6VXX). Residues 581-676, a C-terminal region of the S1 domain involved in S2 interaction, are shown in green. The black square area is magnified on the right. Residues within 5.5 Å of D614 are shown in a ball-and-stick representation. Aspartic acid 614 is shown in bright green. **b** A representation of the SARS-CoV-2 S protein (top) and D/G variation at residue 614 presented in logo plots at different time points between January 1^st^ and May 30^th^, 2020 (bottom). **c**–**e** Mock- and hACE2-293T cells (**c**, **d**) or the same cells transfected to express hTMPRSS2 (**e**) were infected with the indicated PV. See Supplementary Figs. 1a–c and 2 for PV yields, TMPRSS2 expression in transfected HEK293T cells, and the sequences for various FKO mutations, respectively. **f** NCI-H1975 cells transduced to express hACE2 were infected with PVs. See Supplementary Fig. 3a for PV yields. The S proteins used in **c**–**f** have the FLAG tag at C-terminus. Mean values ± SEM of *n* = 5 (**d**), *n* = 3 (**e**), or *n* = 4 (**f**). Experiments conducted using two or three biologically independent PV batches are shown. The *p* values by two-way ANOVA (analysis of variance) with Sidak multiple comparisons test are indicated (**d**, **e**, **f**). *NTD* N-terminal domain, *RBD* receptor-binding domain, *FP* fusion peptide, *HR1* and *HR2* heptad-repeat regions 1 and 2, respectively, *TM* transmembrane region, *CT* cytoplasmic tail*, GFP* green fluorescent protein, *FKO* furin-cleavage knockout mutant, *PV* pseudovirus, *M.F.I.* mean fluorescence intensity.

Recent analyses of the fine-scale sequence variation of SARS-CoV-2 isolates identified several genomic regions of increased genetic variation^21–30^. One of these variations encodes an S-protein mutation, D614G, in the carboxy (C)-terminal region of the S1 domain^21–23,26,30^. This region of the S1 domain directly associates with S2 (Fig. 1a). The mutant virus with glycine at the residue 614 (G614), replacing aspartic acid (D614), has been shown to rapidly dominate in many locales where it is found^21,22^. Our own analyses of the S-protein sequences available from the GenBank showed a similar result: the G614 genotype was not detected in January and February (among 17 and 33 sequences, respectively) and was observed in a minority of sequences in March (26% of 293 sequences), but its frequency increased rapidly by April (65% of 1511 sequences) and May (70% of 2544 sequences) (Fig. 1b). This result suggests a potential transmission advantage of G614 virus over D614 virus, although we cannot exclude the contribution of non-stochastic sampling of virus sequences and random founder effects to its current predominance. At least two groups independently noted that D614G mutation correlated with increased viral loads in COVID-19 patients^22,31^, but because this change is also associated with three or four other mutations in other viral proteins including nsp3 and RdRp, the role of the S protein in these observations remained undefined.

Here we show that the D614G mutation indeed increases pseudovirus (PV) infectivity and that reduced S1 shedding and increased S-protein density in the virion correlate with this increased infectivity.

## Results

### The D614G mutation is associated with enhanced infectivity

To determine if the D614G mutation alters the properties of the S protein in a way that could impact transmission or infection, we assessed its role in viral entry. Maloney murine leukemia virus (MLV)-based PVs, expressing enhanced green fluorescent protein (eGFP) and pseudotyped with the S protein of SARS-CoV-2 (SARS2) carrying the D614 or G614 genotype (S^D614^ and S^G614^, respectively), were produced from transfected HEK293T cells. An S^D614^ variant, in which the furin-cleavage site between the S1 and S2 domains is knocked out (S^FKO^) and generated by replacing the RRAR furin-cleavage motif with SRAS, was also included for comparison. HEK293T cells transduced to express human ACE2 (hACE2-293T) or those transduced with vector alone (Mock-293T) were incubated with PVs pseudotyped with S^D614^, S^G614^, or S^FKO^ (PV^D614^, PV^G614^, or PV^FKO^, respectively), and entry levels were assessed 24 h later. We observed that PV^G614^ entry into the hACE2-293T cells was markedly higher than PV^D614^ entry (Fig. 1c, d). To investigate whether this enhanced entry of PV^G614^ could be affected by TMPRSS2 expression, we conducted similar entry assays in hACE2-293T cells transfected to express hTMPRSS2 (Supplementary Fig. 1c). Entry of PV^G614^ was similarly enhanced relative to PV^D614^ in hACE2-293T cells, with or without TMPRSS2 expression (Fig. 1e). Entry of PV^FKO^ was much more efficient than PV^D614^ (Fig. 1c–e), in contrast to observations from other groups^5,12,32^. This difference is likely explained by the different sequences used to replace the furin-cleavage motif (Supplementary Fig. 2), and suggests that S^FKO^ used in this study is a better substrate for proteases such as TMPRSS2 and cathepsins that cleave the S1/S2 boundary. Because PVs enter ACE2/TMPRSS2-293T cells via both the endosomal and cell-surface pathways, even in case PV^G614^ is less effective in utilizing TMPRSS2 compared to PV^D614^, it may not be detectable. Therefore, we assessed PV entry in a lung epithelial cell line, NCI-H1975, a cell line susceptible to retroviral transduction. Because SARS-CoV-2 target cells in vivo express ACE2^33–35^, we transduced these cells to express hACE2 (Supplementary Fig. 3b, c) and observed PV^G614^ entry was again much more efficient than that of PV^D614^ (Fig. 1f).

The enhanced entry of PV^G614^ into hACE2-293T and lung epithelial cells is not an artifact of differences in PV production, as the yields of PVs measured by RT-qPCR were similar (Supplementary Figs. 1a, b and 3a). Nor it is the result of differential expression of S^D614^ and S^G614^, because the expression of total S protein measured by western blot (WB) in cell lysates and by flow cytometry in transfected cells shows comparable levels for S^D614^ and S^G614^ (Supplementary Fig. 4a, b). Together, these data show that PV^G614^ mediates more efficient entry into ACE2-expressing cells than does PV^D614^.

### Superior infectivity of PV^G614^ is associated with decreased S1 shedding and higher S-protein density in the virion

**Fig. 2:**
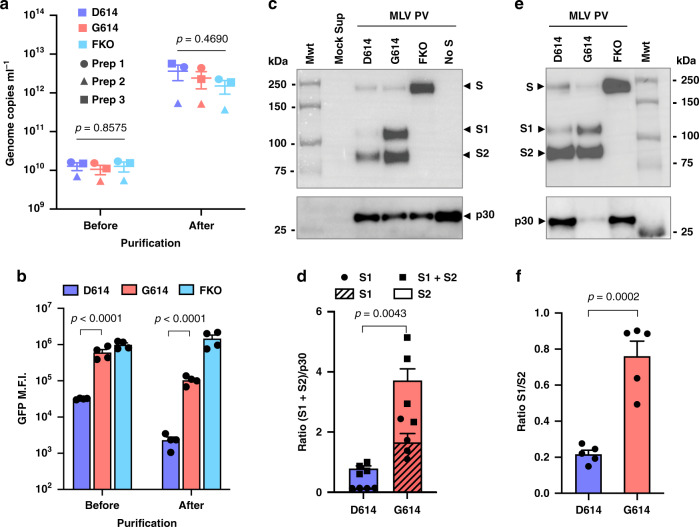
Superior infectivity of PV^G614^ is associated with decreased S1 shedding and higher level of S protein in the virion. **a**–**f** Indicated MLV PVs produced with the S protein containing the FLAG tag at both the N- and C-termini were purified by pelleting through a 20% sucrose layer. PV yields were assessed by RT-qPCR (**a**). The same symbols in different PV groups indicate they are from the same batch. The same PVs were assessed for their infectivity in Mock and hACE2-293T cells (**b**). Mean ± SEM of *n* = 3 biologically independent PV batches (**a**) and *n* = 4 experiments using those three PV batches (**b**) are shown. The same amount (1 × 10^10^ vg per lane) (**c**, **d**) or different amounts to more accurately compare the S1 and S2 ratio (**e**, **f**) of the purified PVs were analyzed by WB (western blot) using the anti-FLAG M2 antibody or anti-p30 MLV gag antibody. The same PVs visualized by silver stain are shown in Supplementary Fig. 5. Total virion S protein (**d**) and the S1:S2 ratio (**f**) of PV^D614^ and PV^G614^ were calculated from *n* = 4 (d) or *n* = 5 (**f**) WBs performed with three independently prepared PV batches and presented as mean ± SEM. The *p* values by one-way ANOVA (**a**), two-way ANOVA with Sidak multiple comparison tests of log-transformed data (**b**), or two-sided unpaired Student’s *t*-test (**d**, **f**) are indicated. *GFP* green fluorescent protein, *MLV* Maloney murine leukemia virus*, PV* pseudovirus, *FKO* furin-cleavage knockout mutant, *M.F.I.* mean fluorescence intensity.

We next investigated the mechanism with which the D614G mutation increased entry of PV^G614^. Because S1 residue 614 is proximal to the S2 domain, we first investigated whether this mutation contributes to the association between the S1 and S2 domains. To detect both the S1 and S2 domains, we used the S-protein constructs bearing the FLAG tag at both their amino (N)- and C-termini. PVs pseudotyped with S^D614^, S^G614^, and S^FKO^ were purified and concentrated by pelleting through a 20% sucrose layer^36^ and evaluated for their yield and entry efficiency. Yields were similar among PV^D614^, PV^G614^, and PV^FKO^ (Fig. 2a), and pelleting the PVs through a sucrose layer did not alter the relative entry efficiency between PV^G614^ and PV^D614^ (Fig. 2b). We analyzed these purified PVs by western blots, detecting the S1 and S2 bands with the anti-FLAG M2 antibody. As shown in Fig. 2c, the most noticeable difference between PV^G614^ and PV^D614^ is markedly higher amounts of the S1 and S2 bands of PV^G614^ compared to PV^D614^. Although Fig. 2a made clear that higher S-protein density in PV^G614^ was not a consequence of higher PV yield, we also confirmed this point by detecting the retroviral protein p30 by western blot (Fig. 2c). The densities of p30 bands were comparable in all PVs, indicating that the higher S-protein incorporation observed with PV^G614^ was due to the different incorporation efficiencies between S^D614^ and S^G614^. Indeed, densitometric analyses showed there was 4.7 times more S1 + S2 band in PV^G614^ compared to PV^D614^ (Fig. 2d). We also observed that the ratio of S1 to S2 band intensity is higher in PV^G614^. To more accurately estimate these differences, different amounts of the same samples were analyzed in order to have comparable S2-band intensity (Fig. 2e). The results of quantification show PV^G614^ has approximately 3.5 times higher S1:S2 ratio compared to PV^D614^ (Fig. 2f). We also directly visualized virion S-protein bands by silver staining (Supplementary Fig. 5). Although S2 bands are masked by an MLV protein of a similar size, S1 bands are well separated. Again, the intensity of the S1 band of PV^G614^ is much stronger than that of PV^D614^, while the p30 bands are comparable, a result consistent with those observed using the anti-FLAG M2 antibody.

### SARS-CoV-2 VLP^G614^ and lung epithelial cell-produced PV^G614^ also exhibit decreased S1 shedding and increased total virion S protein

**Fig. 3:**
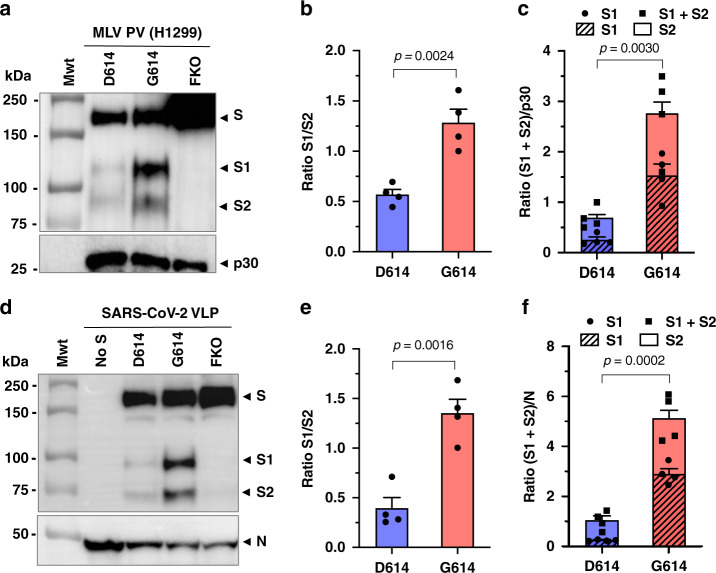
SARS-CoV-2 VLP^G614^ and lung epithelial cell-produced PV^G614^ also exhibit decreased S1 shedding and increased total virion S protein. **a**–**f** PVs produced from NCI-H1299, a lung epithelial cell line (**a**–**c**), and VLPs (virus-like particles) produced from HEK293T cells by transfecting M, N, E, and S proteins of SARS-CoV-2 (**d**–**f**) are analyzed. The S protein is FLAG tagged at both the N- and C-termini. PVs were harvested at 48 h and VLPs at 43 h and purified by pelleting through a 20% sucrose layer. The S protein bands were visualized using the anti-FLAG tag M2 antibody (**a**, **d**), p30 band with an anti-p30 antibody (**a**), and the N protein band using pooled convalescent plasma (**d**). Representative images of WBs (western blot) performed with *n* = 4 independently prepared PV (**a**) or VLP (**d**) batches are shown. The S1:S2 ratio and the difference in total virion S protein incorporation are presented as mean ± SEM, which were calculated from those four PV WBs (**b**,**c**) or four VLP WBs (**e**,**f**). The *p* values by two-sided unpaired Student’s *t*-test are indicated (**b**, **c**, **e**, **f**). *MLV* Maloney murine leukemia virus, *PV* pseudovirus, *FKO* furin-cleavage knockout mutant.

We then explored whether the properties of S^G614^ could be observed when PVs were produced from the lung epithelial cells, emulating SARS-CoV-2 infection of the lung. To produce sufficient amount of PVs, we screened transfection efficiencies of several lung and gastrointestinal epithelial cell lines (Supplementary Fig. 6). Of these, only NCI-H1299 cells were amenable to transfection with low but usable PV yield. Surprisingly, although most SARS-CoV-2 target cells in the lung epithelium in vivo are enriched with furin^33–35^, these cells had little or no furin activity (Supplementary Fig. 6). We thus cotransfected a small amount of furin during PV production in NCI-H1299 cells. These PVs exhibited similar phenotypes as those derived from HEK293T cells (Fig. 3a–c). Thus, as with HEK293T cells, PVs made in a lung epithelial cell line more efficiently incorporated functional S^G614^ compared to S^D614^.

To verify that these observations were not due to properties of retroviral particles, we repeated these studies with virus-like particles (VLPs) composed only of native SARS-CoV-2 proteins, specifically nucleoprotein (N), membrane protein (M), envelope protein (E), and S protein^37^. VLPs were purified and analyzed in the same way as MLV PVs. S-protein bands were detected with the anti-FLAG M2 antibody, and N protein with pooled plasma derived from convalescent COVID-19 patients. As with PVs, the S1:S2 ratio and total S protein on the virion were again much higher in the VLPs carrying S^G614^ (VLP^G614^) compared to those carrying S^D614^ (VLP^D614^) (Fig. 3d–f). Thus, enhanced entry of PV containing the D614G mutation correlates with reduced shedding of the S1 domain and greater incorporation of functional spikes into the virion. To exclude an artifact associated with S-protein tags, we similarly characterized untagged S proteins (Supplementary Fig. 7). Again greater virion incorporation of S protein and higher ratios of S1 to S2 were observed with PV^G614^ compared with PV^D614^.

We next asked if higher stability of PV^G614^ spikes correlated with slower loss of PV infectivity over time. PVs left at room temperature for up to 48 h were used in hACE2-293T entry assays. Under these conditions, we observed no difference in the rate of inactivation among PV^D614^, PV^G614^, or PV^FKO^ (Supplementary Fig. 8a), indicating that loss of infectivity by these PVs is attributed to factors other than differences in their S proteins. This unexpected result suggested the possibility that most S1-domain shedding from S^D614^ occurs before the test period. To evaluate this possibility, we purified PVs from intracellular compartments and observed that S1 shedding had already taken place (Supplementary Fig. 8b). These data show that low stability of S^D614^ did not accelerate PV^D614^ inactivation, because most S1 shedding occurred prior to PV release from the cell.

### The D614G mutation neither increases S protein affinity for ACE2 nor makes PV more resistant to neutralization

**Fig. 4:**
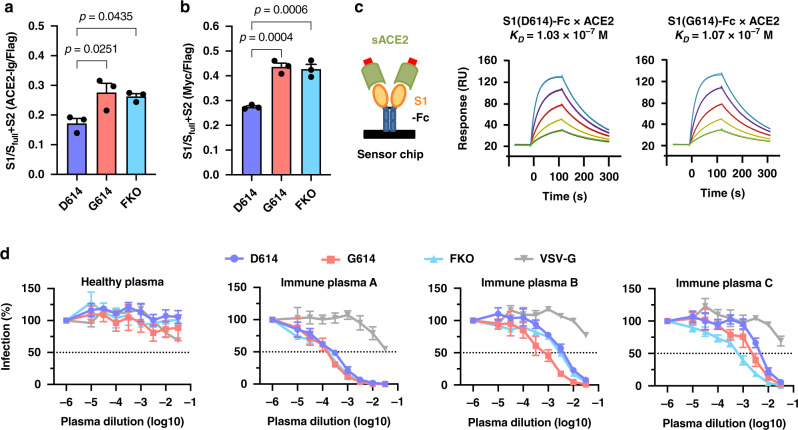
The D614G mutation neither increases S protein affinity for ACE2 nor makes PV more resistant to neutralization. **a** The S protein containing C-terminal FLAG tag is transfected into HEK293T cells and assessed for hACE2-NN-Ig binding. Total S protein was measured by detecting the FLAG tag in the permeabilized cells. The ratio of hACE2-NN-Ig binding to FLAG-tag staining is shown. **b** Experiments similar to those in **a** except that the S protein contains N-Myc and C-FLAG tags, and S1 level was assessed using an anti-Myc antibody. The data in **a**,**b** before normalization are presented in Supplementary Fig. 9a, b. Mean ± SEM of *n* = 3 independent experiments are shown. The *p* values by one-way ANOVA and Sidak multiple comparisons test are indicated. **c** Surface plasmon resonance assay design (left) and sensorgrams (middle and right). S1-Fc was immobilized and monomeric hACE2-NN-Ctag^40^ was injected at 500, 250, 125, 62.5, and 31.25 nM. Colored lines are the experimental traces and black lines are the best global fits (1:1 Langmuir binding model) used to calculate the association (*k*_on_) and dissociation (*k*_off_) rate constants. Representative sensorgrams of three independent experiments with nearly identical results are shown. Supplementary Fig. 9c shows the proteins used in these assays and Fig. 9d presents *k*_on_, *k*_off_, and *K*_*D*_ values derived from *n* = 3 independent experiments. **d** MLV PVs pseudotyped with the indicated S protein (C-term FLAG) or VSV G protein were preincubated without (presented at *x* = −6) or with serially diluted convalescent or control plasmas. hACE2-293T cells were incubated with these preincubated mixes and analyzed 24 h later by measuring luciferase activity. Mean ± SEM of *n* = 3 independent experiments are presented. *FKO* furin-cleavage knockout mutant.

It has previously been speculated that D614G mutation promotes an open configuration of the S protein^22,23^ that is more favorable to ACE2 association^5,38^. To explore this possibility, we investigated whether ACE2 binding to S^G614^-spikes, S-protein trimers, was more efficient than to S^D614^-spikes. HEK293T cells transfected to express each of these S proteins were assessed for their binding of hACE2 immunoadhesin, whose enzymatic activity was abolished by mutation (hACE2-NN-Ig). We previously showed hACE2-NN-Ig bound SARS-CoV-1 RBD with an equivalent affinity as hACE2-Ig^39^. The S proteins used in this study are fused to the FLAG tag at the C-terminus, but not at the N-terminus, thus allowing for the measurement of total S protein expression with an anti-FLAG antibody. As Supplementary Fig. 9a shows, total expression of S^D614^, S^G614^, and S^FKO^, assessed by FLAG-tag staining of permeabilized cells, was comparable. However, hACE2-NN-Ig binding to cells expressing S^G614^- and S^FKO^-spikes was substantially higher than that to cells expressing S^D614^-spikes (Fig. 4a and Supplementary Fig. 9a). There are several explanations for this observation. First, S^G614^ increased ACE2 association by promoting an open configuration of the RBD that was suggested more favorable to ACE2 binding^5,22,23,38^. Second, this mutation actually increased S1 affinity to ACE2, although the residue 614 was outside of the RBD. Third, S^G614^ increased total ACE2 binding by reducing S1-domain shedding. To differentiate these possibilities, we appended the Myc-tag to the N-terminus of the S-proteins that are FLAG tagged at its C-terminus and repeated the experiment, this time detecting the S1 domain using an anti-Myc antibody. As Supplementary Fig. 9b shows, while total expression of S^D614^, S^G614^, and S^FKO^, assessed by FLAG-tag staining of permeabilized cells, is similar, the levels of S^G614^- and S^FKO^-spikes on the cell surface, measured by an anti-Myc antibody, were considerably higher than that of S^D614^-spikes. In fact, the ratio of Myc tag to FLAG tag is similar to the ratio of hACE2-binding to FLAG tag (Fig. 4a, b), indicating that increased ACE2 binding to the S^G614^-spikes did not result from its increased affinity to S^G614^ or the open RBD configuration. Instead, these data indicate that a higher number of the S1 domain is present on the surface of S^G614^-expressing cells. To confirm that the monomeric interaction between hACE2 and S protein is not affected by the D614G mutation, we performed surface plasmon resonance assays. Soluble monomeric hACE2-NN bound with a nearly identical affinity to S1^G614^-Fc and S1^D614^-Fc immobilized on a chip (Fig. 4c), a result that is expected because the residue 614 is outside of the RBD. Together, these results indicate that increased ACE2 binding to the cell surface-expressed S^G614^ spikes is due to decreased S1 shedding.

Finally, we assessed whether differential amount of functional S protein on the virion could influence neutralization sensitivity of the virus. Fig. 4d shows that PV^D614^, PV^G614^, and PV^FKO^ are similarly susceptible to neutralizing plasma derived from convalescent patients, indicating that antibody-mediated control of viruses carrying S^D614^ and S^G614^ would be similar, and that the vaccines based on the D614 genotype will still be effective against the virus carrying the D614G mutation.

## Discussion

Our data make clear that there are consistent differences in the efficiency with which S^D614^ and S^G614^ are incorporated into PVs and VLPs. However, the physical basis for these differences remains undefined. The different ratios of S1:S2 suggest that association between these two domains is weaker in S^D614^. The apparent instability of S^D614^ could impact the integrity of the local membrane, appropriate trafficking of the S protein, or appropriate post-translational modification of the S-protein cytoplasmic tail, which may in turn impact S-protein incorporation into the virion. Our data also make clear that both a functional proprotein convertase and a functional furin-cleavage motif are necessary to observe differences between S^D614^ and S^G614^.

Although evidence is still accumulating, the increasing predominance of D614G in humans raises the possibility that viruses with this mutation have a fitness advantage, perhaps allowing more efficient person-to-person transmission. Our virological data are consistent with, but do not themselves demonstrate, this hypothesis. Interestingly, this mutation does not appear to significantly impact disease severity^22,27^. It is possible that higher levels of functional S protein observed with PV^G614^ increase the chance of host-to-host transmission, but other factors limit the rate and efficiency of intra-host replication. Alternatively, the loss of virion-associated S proteins observed with S^D614^ may be compensated by greater fusion efficiency with the destabilized S protein when the next target cell is adjacent in a host tissue. It is also possible that our ability to detect the emergence of new mutations at this stage of the pandemic is simply greater than our ability to detect modest differences in pathogenesis. The strong phenotypic difference we observe here between D614 and G614 suggests that more studies on the impact of the D614G mutation on the course of disease may be warranted.

Finally, our data raise interesting questions about the natural history of SARS-CoV-2 as it moved presumably from horseshoe bats to humans. At some point in this process, the virus acquired a furin-cleavage site, allowing its S1/S2 boundary to be cleaved in virus-producing cells. In contrast, the S1/S2 boundary of SARS-CoV-1, and indeed all SARS-like viruses isolated from bats, civet cats, and pangolins lack this polybasic site and are instead cleaved by TMPRSS2 or endosomal cathepsins in the target cells^13–20^. As we have shown in this study, there are two mechanisms with which SARS-CoV-2 could have enhanced its infectivity, namely, through D614G mutation or elimination of furin-cleavage site with an optimized replacement (Supplementary Fig. 2). However, as the virus transmits in humans, it has clearly retained the furin site while compensating for this site with D614G mutation. Therefore, the furin-cleavage site itself appears to confer a yet-to-be-determined fitness advantage.

In summary, an S-protein mutation, D614G, that appears to promote SARS-CoV-2 transmission in humans also enhances functional S-protein incorporation into SARS-CoV-2 VLP and retroviral PV and increases PV infectivity. Further studies will be necessary to determine the impact of this change on the nature and severity of COVID-19.

## Methods

### SARS-CoV-2 S protein sequences analysis

To track the D614G variation among SARS-CoV-2 isolates, S protein sequences were downloaded from GenBank by searching for “SARS-CoV-2 spike” and by filtering the result based on sequence length “1272–1273” to retrieve full-length S protein. To separate retrieved sequences by the month, the result was further filtered by release date. Genotype frequency at residue 614 was calculated using R version 3.6.0 (R Foundation for Statistical Computing) with the Biostrings package version 2.52. Logo plots of D614G variation were generated by WebLogo version 3 after sequence alignment. Total number of sequences analyzed for each month is 17 sequences in January, 33 in February, 293 in March, 1511 in April, and 2544 in May.

### MLV PV production

PV production from HEK293T cells: Cells at ~60% confluence in T75 flasks were transfected by the calcium-phosphate method with 24 μg of total DNA at a ratio of 5:5:1 by mass of the retroviral vector pQCXIX encoding enhanced green fluorescence protein (eGFP) or firefly luciferase (FLuc), a plasmid expressing MLV gag and pol proteins, and a plasmid expressing the S protein of SARS-CoV-2. SARS-CoV-2 S protein gene was codon-optimized and synthesized by Integrated DNA Technologies based on the protein sequence (GenBank YP_009724390), and cloned in pCAGGS vector. The S protein gene was fused or not to the FLAG tag sequence either at its C-terminus or at both the N- and C-termini, as indicated in each experiment. More information on the plasmids used in the study is provided in Table 1. Transfected cells were washed at 6 h post transfection (hpt) and replenished with 10 ml DMEM supplemented with 10% FBS. PV-containing culture supernatants were collected at 43 hpt, cleared through 0.45 μm filters, and either purified or aliquoted and frozen at −80 °C immediately.

**Table 1:** Plasmids used in the study.

Protein	Modification	Note
hACE2	N-terminal Myc and C-term c9 tag (TETSQVAPA)	mouseACE2 signal seq + Myc + hACE2 residues 20-805 (NM_021804)
hTMPRSS2	C-terminal FLAG	Full-length (NP_005647.2)
hFurin	C-term c9 tag	Full-length (BC012181.1)
S1^D614^-Fc	C-terminal Fc	Residues 1-682; codon-optimized (YP_009724390)
S1^G614^-Fc	C-terminal Fc	Residues 1-682; codon-optimized; D614G mutation
hACE2-NN-Ig	C-terminal Fc	IGH3 signal seq + hACE2 residues 20-615 (NM_021804) with H374N and H378N mutations
hACE2-NN-Ctag	C-terminal Ctag (-EPEA)	IGH3 signal seq + hACE2 residues 20-615 (NM_021804) with H374N/H378N mutations
MLV gag/pol	None	Full length MLV gag and pol
pQCXIX-eGFP	None	Full-length eGFP in retroviral vector pQCXIX
pQCXIX-FLuc	None	Full-length firefly luciferase in pQCXIX
S protein (D614, G614, or FKO)	C-terminal FLAG	Full-length; codon-optimized (YP_009724390)
	N-/C-term FLAG	Full-length; codon-optimized; its own signal sequence
	No tag	Full-length; codon-optimized
M protein	None	Full-length; codon-optimized (YP_009724393)
N protein	None	Full-length; codon-optimized (YP_009724397)
E protein	None	Full-length; codon-optimized (YP_009724392)

PV production from NCI-H1299 cells: NCI-H1299 cell line was kindly provided by Joseph Kissil (The Scripps Research Institute, Jupiter, FL, USA), authenticated by ATCC Cell Line Authentication Service, and cells were maintained in RPMI supplemented with 10% FBS. Cells at ~90% confluence in T75 flasks were transfected with total 24 μg DNA using FuGENE (Promega) at a ratio of 1:3 of DNA:FuGENE. Because of low furin activity in these cells, 2 μg furin expression plasmid (Table 1) was cotransfected. The remaining 22 μg DNA was split at a 5:5:1 ratio among retroviral vector pQCXIX encoding eGFP, a plasmid expressing MLV gag and pol proteins, and a plasmid expressing the S protein fused to N- and C-terminal FLAG tag. S^D614^, S^G614^, and S^FKO^ (Supplementary Fig. 2) were used. Cells were split by trypsinization into two T75 flasks at 6 hpt, and replenished with 10 ml RPMI per flask supplemented with 10% FBS. Culture supernatants containing PVs were collected at 48 hpt, cleared of cell debris through centrifugation at 3000 ×*g* for 10 min, and subjected to purification.

PV isolation from intracellular compartments: Transfected cells on a T75 flask were collected, washed once with PBS, and resuspended in 2 ml PBS containing protease inhibitor (Thermo Scientific, A32955). Intracellular PVs were released from three cycles of freezing and thawing, and cell debris was removed by centrifugation for 10 min at 3000 ×*g*. Supernatants were cleared by 0.45 μm filtration, the volume was increased to 9 ml with PBS, and subjected to purification.

### PV purification

Here, 9 ml of cleared culture supernatants containing PVs were loaded onto 2 ml of 20% sucrose (Signa-Aldrich, S7903) in NT buffer (120 mM NaCl, 20 mM Tris, pH 8.0) and centrifuged at 30,000 rpm in the SW41 rotor (154,000 ×*g*) for 2 h at 10 °C^36^. The pellets of PVs derived from HEK293T cells were resuspended in 30–50 μl NT buffer with gentle shaking on ice and either immediately used or aliquoted and frozen at −80 °C. The pellets of PVs derived from NCI-H1299 cells were difficult to resuspend, likely because of mucin secreted by lung epithelial cells, and thus resuspended in 30 μl NT buffer containing 0.5% SDS with gentle shaking at room temperature, avoiding bubbling, and used only in WB analyses.

### PV quantification

PVs were quantified by RT-qPCR, using primers and a probe that target the CMV promoter. Culture supernatants containing PVs were treated with 100 μg/ml RNase A for 1 h at 37 °C to degrade RNAs that are not packaged inside the virion, and RNA was extracted with Trizol and GlycoBlue coprecipitant and digested for 30 min at 37 °C with DNase I at 1 IU per 1 μg extracted RNA. DNase I was inactivated by incubating for 10 min at 65 °C with EDTA added to final 5 mM. DNase-treated RNA was reverse transcribed using the High-Capacity cDNA Reverse Transcription Kit (Applied Biosystems, 4374966). qPCR was performed, using Luna Universal Probe qPCR Master Mix (New England Biolabs, M3004E) with the known quantity of pQCXIX vector to generate standard curves and data were collected with CFX Manager 3.1 (Bio-Rad). Primers and a probe were synthesized at Integrated DNA Technologies. Sense primer: 5ʹ-TCACGGGGATTTCCAAGTCTC-3ʹ, anti-sense primer: 5ʹ-AATGGGGCGGAGTTGTTACGAC-3ʹ, probe: 5ʹ-FAM-AAACAAACT-(ZEN)-CCCATTGACGTCA-IBFQ-3ʹ.

### PV entry assay in HEK293T cells

PV entry assays in HEK293T cells were performed by spinoculating PVs (5 × 10^8^ genome copy/well) onto the Mock-293T, hACE2-293T, or hACE2-293T cells transfected to express hTMPRSS2 seeded on 48-well plates one day before. Spinoculation was carried out at 2100  × *g* for 30 min at 25 °C. Spinoculated plates were incubated for 2 h in a CO_2_ incubator and medium was replaced with DMEM containing 10% FBS. Entry levels in GFP expression were measured 24 h later in the viable cell population gated in pseudocolor plots by Accuri flow cytometer using C6 software 1.0.264 (BD) and ForeCyt 6.2R3 (IntelliCyt). An example of gating strategy is shown in Supplementary Fig. 10. Entry levels in luciferase activity was assessed using the Luc-Pair Firefly Luciferase HS Assay Kit (GeneCopoeia, LF009) and data were collected with SoftMax Pro 6.3 (Molecular Devices).

### ACE2 expression and PV entry assay in lung epithelial cell lines

Cells: Calu-3 was recently purchased from ATCC and grown in EMEM supplemented with 10% FBS. NCI-H1299, NCI-H1975 were kindly provided by Joseph Kissil (The Scripps Research Institute, Jupiter, FL, USA) and maintained in RPMI supplemented with 10% FBS. NCI-H1299 and NCI-H1975 were authenticated using Short Tandem Repeat analysis through ATCC Cell Line Authentication Service. Cells were grown to approximately 70% confluence before splitting by trypsinization.

Assessment of ACE2 expression in lung epithelial cell lines: Calu-3, NCI-H1299, and NCI-H1975 cells on 6-well plates were detached by scraping in 0.5 ml of PBS containing 5 mM EDTA. hACE2-293T cells were used as a positive control. For hACE2-transduced Calu-3 and NCI-H1975 cells, staining was performed 2 days post transduction. Approximately 0.5 × 10^6^ cells per well in V-bottom 96-well plates were incubated on ice for 45 min with 3 μg/ml anti-hACE2 antibody (R&D, MAB9332) in PBS containing 2% goat serum (Gibco, 16210064), followed by goat-anti-mouse IgG conjugated with phycoerythrin (Jackson ImmunoResearch, 115-116-146) diluted at 1:500 in PBS containing 2% goat serum. Cells were washed twice with PBS containing 2% goat serum and once with PBS, fixed with 2% paraformaldehyde in PBS, and viable cell population gated in pseudocolor plots was analyzed by Accuri flow cytometer using C6 software 1.0.264 (BD) and ForeCyt 6.2R3 (IntelliCyt).

PV entry assay: NCI-H1975 cells were transduced to express hACE2 and seeded next day on 48-well plates to achieve ~30% at the time of an entry assay. Two days post transduction, cells were spinoculated with PVs (5 × 10^8^ genome copy/well) at 2100 × *g* for 30 min at 25 °C. Spinoculated plates were incubated for 2.5 h in a CO_2_ incubator, and medium was replaced. Entry levels were assessed 48 h post infection by measuring GFP expression in the viable cell population gated in pseudocolor plots by Accuri flow cytometer using C6 software 1.0.264 (BD) and ForeCyt 6.2R3 (IntelliCyt).

### Analysis of S1 shedding and S protein density of MLV PV

For the analyses of S1 shedding and S-protein density on the virion, purified PVs were analyzed by SDS-PAGE and WB analyses. Approximately 1/10 of purified PVs or VLPs derived from one T75 flask of transfected HEK293T cells or all of purified PVs from two T75 flasks of NCI-H1299 cells (see PV production) were analyzed in each WB analysis.

PV proteins were separated on the 4-12% Bis-Tris gel (Life Technologies, NW041122), transferred to the PVDF membrane, and blotted with 1 μg/ml anti-FLAG M2 antibody (Sigma-Aldrich, F1804) to detect the S-protein bands or 1 μg/ml anti-p30 MLV gag antibody (Abcam, ab130757) to detect p30 bands as an PV quantity control. Bands were visualized with 1:10,000 diluted goat-anti-mouse IgG-HRP polyclonal antibody (Jackson Immuno Laboratory, 115-036-062) and SuperSignal West Atto Ultimate Sensitivity Substrate (Thermo Scientific, A38555). Band intensities were measured using Image Lab software (Bio-Rad). To increase the accuracy of this measurement, the same blots were analyzed several times at different exposures. Although PV^D614^ and PV^G614^ containing the same amount of S2 domain and p30 gag would have been useful to confirm differential S1 shedding by these PVs, generating such PVs is technically impossible, owing to the built-in property of S^G614^ to incorporate into the virion with much higher efficiency than does S^D614^.

For silver staining, all of the purified PVs from one T75 flask of HEK293T cells were separated by the 4–12% Bis-Tris gel and stained with Silver Stain Plus kit (Bio-Rad, 1610449). For the PVs produced with the S protein lacking a tag, the S1 and S2 bands were detected using convalescent plasma at 1:250 dilution followed by 10 ng/ml mouse-anti-human IgG antibody conjugated with polymerized HRP (Fitzgerald, 61R-I166AHRP40) and SuperSignal West Atto Ultimate Sensitivity Substrate.

### SARS-CoV-2 VLP production and S-protein analysis

SARS-CoV-2 VLPs were produced by transfecting HEK293T cells at ~60% confluence in T75 flasks with 25 μg total DNA using the calcium phosphate method. The plasmids expressing SARS-CoV-2 M, N, E, and S proteins were transfected at a ratio of 1:5:5:1. The codon-optimized M, N, and E protein genes were synthesized based on the GenBank protein sequences, YP_009724393, YP_009724397, and YP_009724392, respectively. The S proteins with the FLAG tag at both the N- and C-termini were used. Transfected cells were washed at 6 hpt and VLPs were harvested 43 hpt from the culture supernatants, cleared by 0.45 μm filtration, and purified by pelleting through a 20% sucrose layer as were MLV PVs. VLP pellets were resuspended in 30 μl of NT buffer, and the entire amount derived from one T75 flask was analyzed by SDS-PAGE and WB analysis. The S-protein bands were detected using the anti-FLAG M2 antibody, and the N-protein bands were detected using pooled convalescent plasma at a 1:500 dilution. Bands were visualized with 10 ng/ml mouse-anti-human IgG antibody conjugated with polymerized HRP (Fitzgerald, 61R-I166AHRP40) and SuperSignal West Atto Ultimate Sensitivity Substrate.

### Cell-surface expression and analysis of the S protein

HEK293T cells, approximately 80% confluent in 6-well plates were transfected with 8 μl PEI 40,000 (Polysciences) and 2 μg plasmid expressing the indicated S protein variant. For ACE2-NN-Ig binding experiments, the S protein constructs with the FLAG tag at the C-terminus were used. NN denotes the mutations of two histidines to asparagines at the positions 374 and 378, which inactivate ACE2 enzyme activity but do not affect S-protein binding^39^. To measure S1 and total S protein, the S proteins with N-terminal Myc tag and C-terminal FLAG tag were used. All tags were fused with a two-glycine (GG) linker. To measure ACE2-binding ability or the level of the S1 domain present on the cell surface, cells were detached two days post transfection with Accutase (Stemcell Technologies Inc.) and approximately 5 × 10^5^ cells were incubated on ice in 100 μl of either 1 μg/ml purified hACE2-NN-Ig followed by 1:200 diluted anti-hIgG-FITC (Jackson ImmunoResearch, 109-096-097) or 3 μg/ml anti-Myc antibody (clone 9E10, National Cell Culture Center, Minneapolis, MN) followed by 1:200 diluted anti-mIgG-APC (Jackson ImmunoResearch, 115-136-072), respectively. hACE2-NN-Ig was purified using Protein A-Sepharose CL-4B (GE Healthcare). To measure total level of the S protein, cells were permeabilized with 0.5% Triton X-100 (Sigma-Aldrich) in PBS for 10 min at room temperature and incubated with 1 μg/ml anti-FLAG M2 antibody (Sigma-Aldrich, F1804) followed by 1:200 diluted anti-mIgG-APC (Jackson ImmunoResearch, 115-136-072).

### Surface Plasmon Resonance (SPR) analysis

The proteins used in SPR analyses were produced from transient transfection of Expi293 (Thermo-Fisher) using FectoPRO (Polyplus) with plasmids encoding the Fc-fusion forms of SARS-CoV-2 S1 domain (S1-Fc, residues 1-682) or hACE2-NN (residues 20-615) with a C-terminal Ctag (-EPEA)^40^. After 5 days in shaker culture, media were collected and cleared of debris by low-speed centrifugation and 0.45-µm filtration. S1-Fc was purified using MabSelect Sure (GE Lifesciences) and hACE2-NN-Ctag using CaptureSelect C-tagXL (Thermo-Fisher) columns according to the manufacturer’s instruction. The buffer of eluates was exchanged with PBS and concentrated using Amicon ultra filtration devices (Millipore Sigma), and proteins were stored at 4 °C and used within a few days.

SPR measurements were performed on a Biacore X100 instrument at 25 °C using 1x HBS-EP + (Cytiva) as running buffer. A mouse anti-human IgG C_H_2 monoclonal antibody (Cytiva) was immobilized to a density of ~3800 response units (RU) on a CM5 sensor chip via standard NHS/EDC coupling methods (Cytiva). Subsequently, S1^D614^-Fc or S1^G614^-Fc, each at 10 μg/mL, was captured to ~680 RU on the active flow cell. A concentration series with two-fold dilutions (500–31.25 nM) of monomeric hACE2-NN-Ctag were injected using a multi-cycle method. The lowest concentration (31.25 nM) was repeated to confirm regeneration of the sensor chip. Biacore X100 Control Software 2.0.1 (Cytiva) was used to collect data and Biacore X100 Evaluation Software 2.0.1 (Cytiva) to analyze data. Calculation of association (*k*_on_) and dissociation (*k*_off_) rate constants was based on a 1:1 Langmuir binding model. All fitting data revealed a Chi² value of <1. The equilibrium dissociation constant (*K*_*D*_) was calculated from *k*_off_*/k*_on._

### Neutralization assay with human immune plasma

Deidentified blood samples were obtained by the Allergy, Asthma and Immunology Specialists of South Florida, LLC for COVID-19 serotyping, and exempt (IRB-20-7580) from human subject research CFR 45.101(b)(4). MLV PVs encoding firefly luciferase and pseudotyped with the indicated SARS-CoV-2 S protein were preincubated for 1 h at 37 °C with or without convalescent or healthy plasma serially diluted in DMEM containing 10% FBS. Preincubated samples were added to Mock- and hACE2-293T cells on 96-well plates and entry levels were assessed 24 h later by measuring luciferase activity using the Luc-Pair Firefly Luciferase HS Assay Kit (GeneCopoeia, LF009) and data collected with SoftMax Pro 6.3 (Molecular Devices).

### Statistical analysis

All appropriate data were analyzed with GraphPad Prism version 7 (GraphPad Software Inc.). All hypothesis tests were performed as two-tailed tests. Specific statistical analysis methods are described in the figure legends where results are presented. Values were considered statistically significant for *p* values below 0.05. Exact *p* values are provided in appropriate figures.

## Reagent availability

Plasmids and cell lines used in this study are available from the corresponding author upon request.

### Reporting summary

Further information on research design is available in the Nature Research Reporting Summary linked to this article.

## Supplementary information

Supplementary Information

Peer Review File

Reporting Summary

## Data Availability

Source Data are provided with this paper and all relevant data are available from the authors. The data used to generate the Cryo-EM images in Fig. 1a are available from PBD entry 6VXX (http://www.rcsb.org/structure/6vxx). Sequences used to generate logo plots in Fig. 1b are available from GenBank (https://www.ncbi.nlm.nih.gov/protein) by searching for “SARS-CoV-2 spike” and filtering the result by the length of the protein (1272-1273). To separate the result by the month, retrieved sequences can be further filtered by the release date. Source data are provided with this paper.

## Source data

Source Data
