# Supplementary material for: SARS-CoV-2 spike-protein D614G mutation increases virion spike density and infectivity
Source: Nat Commun. 2020 Nov 26;11:6013. doi: 10.1038/s41467-020-19808-4 (PMC7693302; doi:10.1038/s41467-020-19808-4)
Supplement: Supplementary file 1 — Supplementary Information [file 41467_2020_19808_MOESM1_ESM.pdf]

Supplementary Information

SARS-CoV-2 spike-protein D614G mutation increases virion spike density and infectivity

Zhang et al.

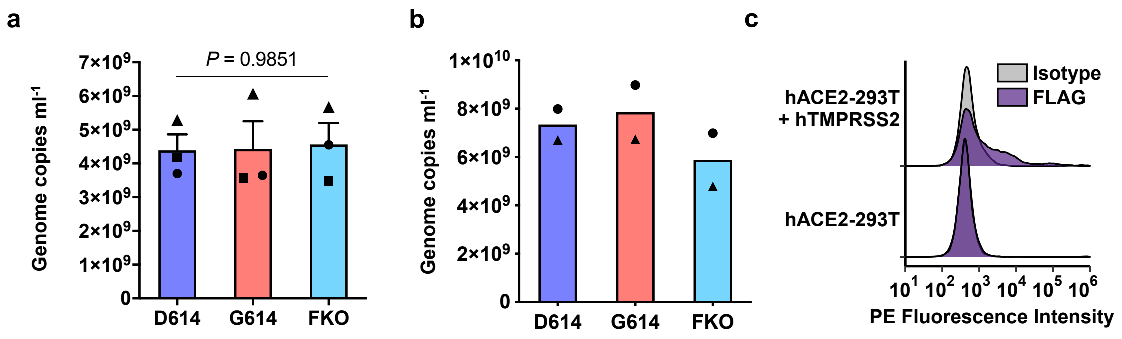

Supplementary Figure 1. The D614G mutation is associated with enhanced infectivity. a,b, The yields of the MLV PVs used in the experiments shown in Fig. 1c,d (a) and Fig. 1e (b) were quantified by RT-qPCR. The same symbols in different PV groups indicate they were from the same batch. Comparable PV yields validate that enhanced infectivity of PV^{G614} did not result from a large difference in virus yields and/or normalization thereof. Mean ± SEM of n=3 (a) or n=2 (b) independent PV preparations are shown. The *p* values by one-way ANOVA are indicated in (a) and (b). **c,** hACE2-293T cells transfected to express hTMPRSS2 were stained for the FLAG tag fused at the C-terminus of TMPRSS2, a type II transmembrane protein. These cells were used in the infection experiments shown in Fig. 1e.

```
WT SARS-COV-2:  QTNSPRRARSV
     Our FKO :  QTNSPSRASSV
FKO, PMID 32376634:  QSLL----RSV
FKO, PMID 32362314:  QTNSP---ASV
FKO, PMID 32155444:  QTIL----ASV
```

Supplementary Figure 2. S-protein furin-cleavage-site mutations used in this and other studies. The furin-cleavage motif of the wild-type SARS-CoV-2 S protein (RRAR) is mutated to different sequences by us (in blue) and by other groups (in orange). Hyphens denote deletion. PV^{FKO} generated by other groups generally showed lower infectivity than wild-type SARS-CoV-2 PV (D614) in various cells. On the other hand, our PV^{FKO} entered hACE2-293T and hACE2-hTMPRSS2-293T cells with higher or much higher efficiency than PV^{G614} or PV^{D614}, respectively (Fig. 1c-e). These results suggest that our FKO sequence might be a better substrate for proteases including TMPRSS2 and cathepsins than those used to make other FKO mutants.

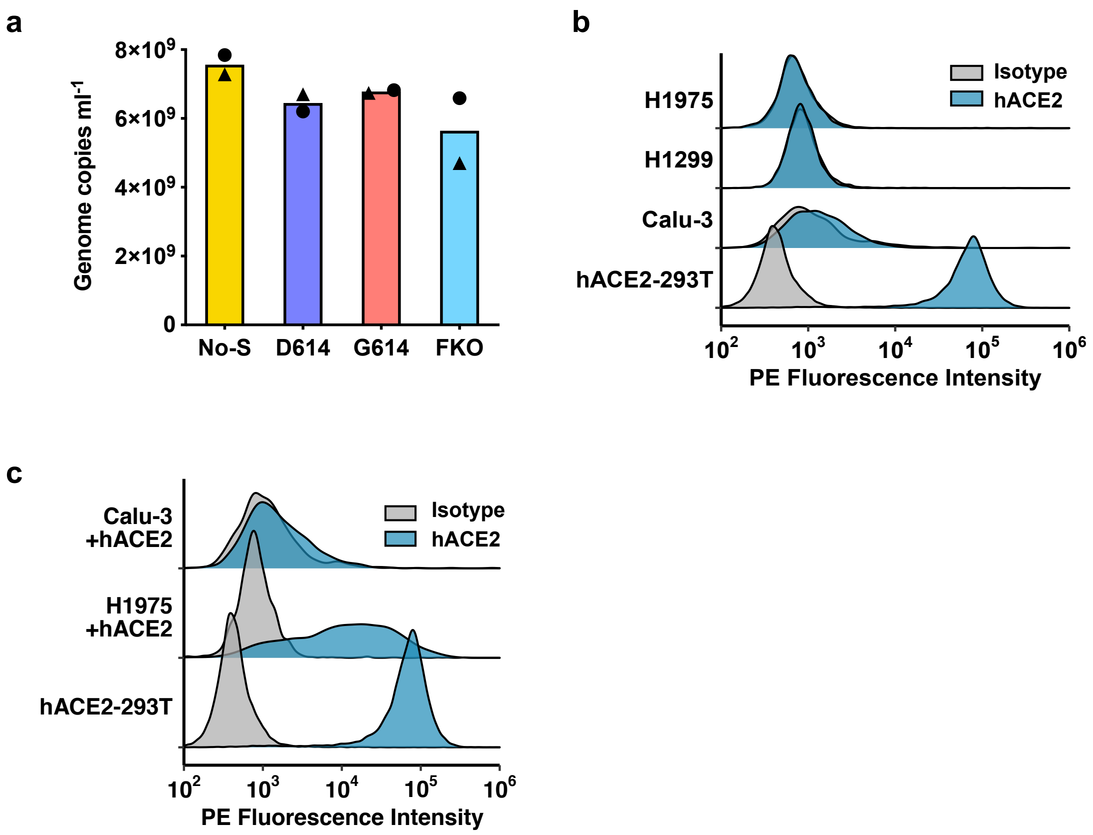

Supplementary Figure 3. Common lung epithelial cell lines express little or no ACE2. a, The yields of the MLV PVs used in the experiments shown in Fig. 1f were quantified by RT-qPCR. The same symbols in different PV groups indicate they were from the same batch. Each symbol indicates an average value of a duplicated assay. Mean ± SEM of n=2 independent PV preparations are shown. **b,** Endogenous ACE2 expression in lung epithelial cells. **c,** ACE2 expression levels in Calu-3 or NCI-H1975 cells transduced to express hACE2. Note that Calu-3 is not susceptible to transduction. Transduced NCI-H1975 cells were used in the PV entry assays shown in Fig. 1f. The same hACE2-293T controls are displayed in (b) and (c) because the experiments were performed together.

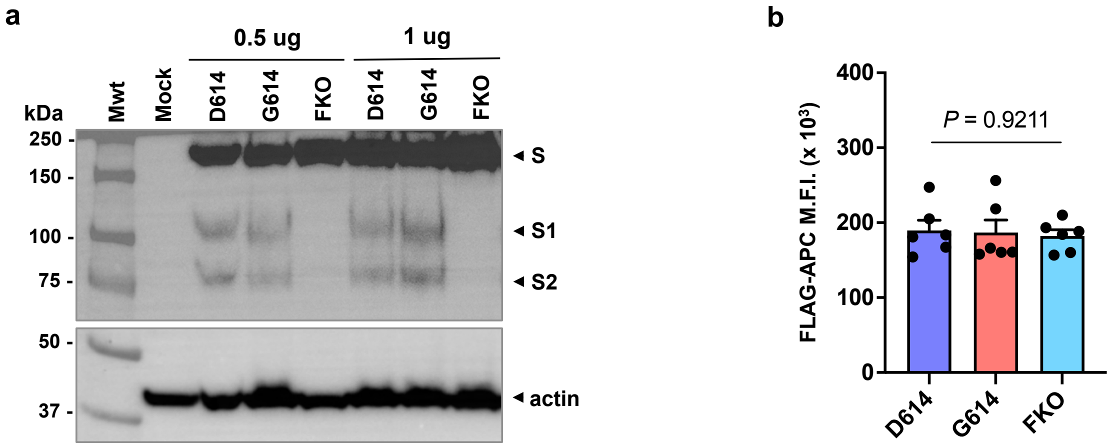

Supplementary Figure 4. S^{D614} and S^{G614} express at comparable levels. a, HEK293T cells on 6-well plates were transfected with 0.5 or 1 μg plasmid to express the indicated S protein and analyzed 48 h later for S-protein expression by WB. The S-protein and actin bands were visualized using the anti-FLAG M2 or anti-actin antibody, respectively. The S proteins used in this experiment have the FLAG tag at both the N- and C-termini. A representative image of two independent experiments is shown. **b,** HEK293T cells transfected to express the indicated S proteins were analyzed 48 h later for S-protein expression by flow cytometry. The S proteins used in this experiment have the FLAG tag only at the C-terminus. Total S protein expression was measured in permeabilized cells, using the anti-FLAG M2 antibody. Mean ± SEM of n=6 independent experiments are shown. Each dot indicates an average value of a duplicated experiment. The *p* value by one-way ANOVA is indicated. M.F.I.: mean fluorescence intensity.

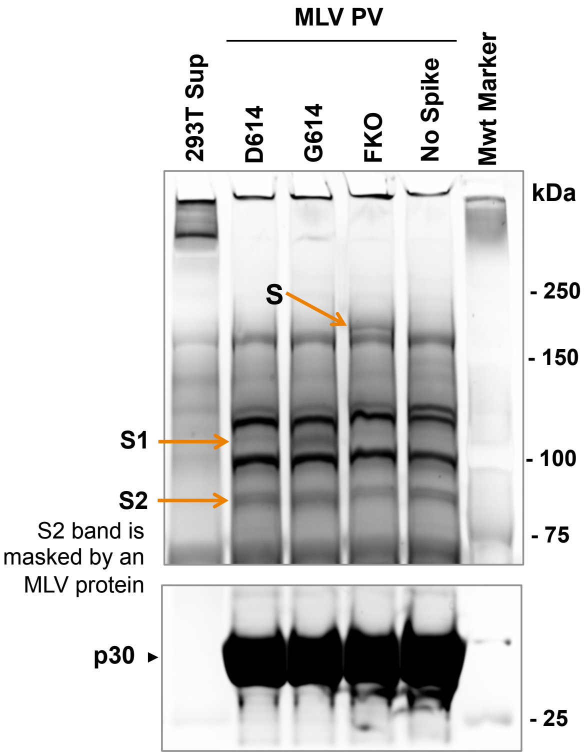

Supplementary Figure 5. Superior infectivity of PV^{G614} is associated with decreased S1 shedding and higher level of S protein in the virion. The same PVs used in the experiment shown in Fig. 2c were analyzed by SDS-PAGE and silver stained to avoid a potential bias caused by using the M2 antibody that recognizes N- and C-terminal FLAG tags with different affinity. Although the S2 band is masked by an MLV-derived protein, the S1 band is clearly separated and much weaker in PV^{D614} compared to that in PV^{G614}. One representative gel is shown from n=2 independent experiments conducted with biologically independent PV batches with similar results.

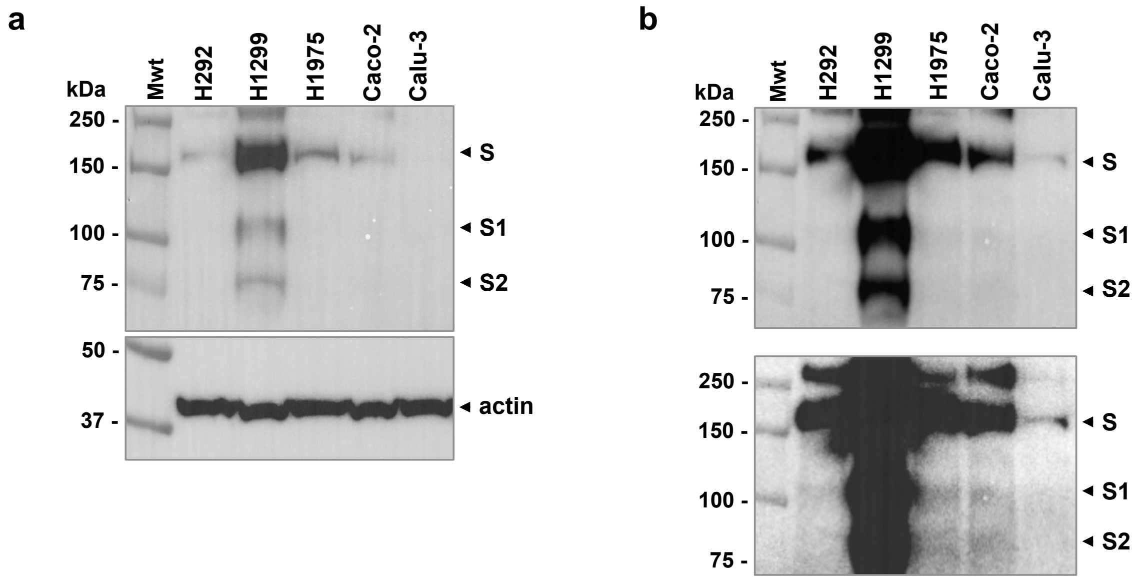

Supplementary Figure 6. Most well-established lung epithelial cell lines have little or no furin activity. a, Indicated cells were seeded on 6 well plates and transfected with 1.5 µg plasmid expressing the S protein (D614) and Fugene at a 1:3 ratio. Cells were lysed at 42 hpt in 200 µl lysis buffer (150 mM NaCl, 50 mM Tris pH7.4, 1% Triton X-100, protease inhibitors, phosphatase inhibitors). 20 µl of each lysate was analyzed by SDS-PAGE and WB, using the anti-FLAG M2 antibody and an anti-actin antibody. Except NCI-H1299, all cells were hard or very hard to transfect and exhibit poor S-protein cleavage, indicating low expression of proprotein convertases including furin. One representative blot is shown from n=2 independent experiments with similar results. **b,** A longer (top) or much longer (bottom) exposure of the same blot shown in (a) to visualize efficiency of S-protein cleavage in these cells.

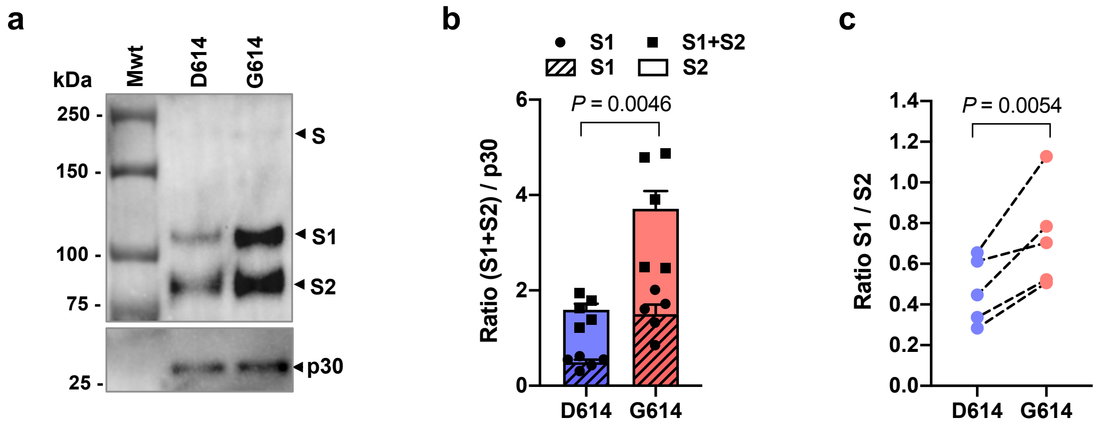

Supplementary Figure 7. S-protein tagging does not substantially impact D614G mutation phenotype. a, PV^{D614} and PV^{G614} were produced with the S proteins containing no tag and purified by pelleting through a 20% sucrose layer. PVs were analyzed by SDS-PAGE and WB. S protein bands were detected with convalescent plasma and p30 with an anti-p30 MLV gag antibody. A representative image of n=5 WB analyses performed with five biologically independent PV batches. **b,c,** The ratios of total virion S protein (b) and S1:S2 (c) were calculated from these n=5 WBs and presented as mean ± SEM. The *p* values by two-way ANOVA with unpaired Student's t-test (b) and ratio paired t test (c) are indicated.

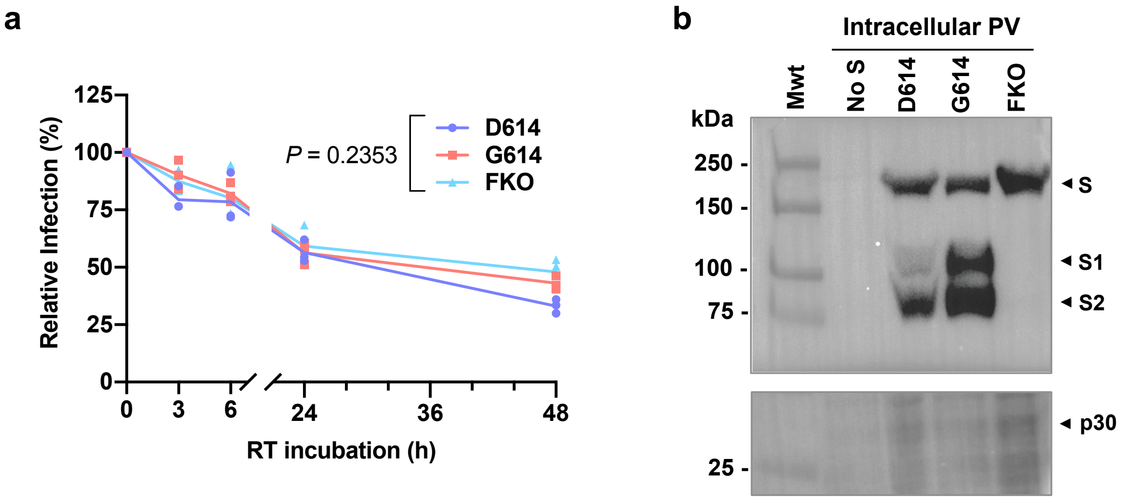

Supplementary Figure 8. S1 shedding occurs early in virion assembly. a, MLV PVs expressing firefly luciferase were produced from HEK293T cells, and aliquots were frozen at -80ºC. Individual aliquots were thawed and left at room temperature (24ºC) for 0, 3, 6, 24, or 48 h before assessed in hACE2-293T cells for their residual infectivity. To compare PVs at similar entry levels, 2×10^8 genome copies (gc) PV^{D614}, 3×10^7 gc PV^{G614}, and 2×10^7 gc PV^{FKO} were used at each time point. Luciferase activity was measured 24 hours later. Shown are the results of n=3 independent experiments performed with two biologically independent batches of PVs. Each dot indicates an average value of a duplicated experiment. Statistical significance was assessed by 2-way ANOVA. Note that although all PVs lost infectivity with time, inactivation of PV^{D614} was not accelerated compared to PV^{G614} or PV^{FKO}, indicating PV inactivation in this condition was contributed by factors other than the S protein. **b,** An analysis of intracellular PVs. HEK293T cells were pelleted at 43 h post transfection and resuspended in 2 ml PBS. One representative blot is shown from n=2 independent experiments with similar results. Intracellular PVs were released via three cycles of freezing and thawing in the presence of a protease inhibitor cocktail, cleared by 0.45 μm filtration, purified by ultracentrifugation through a layer of 20% sucrose, and analyzed by WB. The S protein bands were visualized using the anti-FLAG tag M2 antibody. Note that S1 shedding has already occurred in intracellular virions.

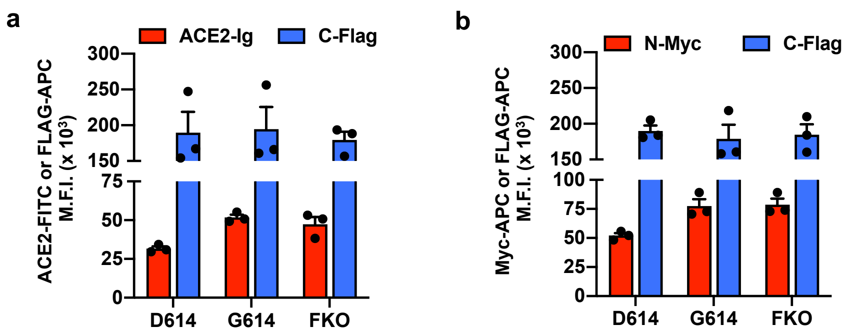

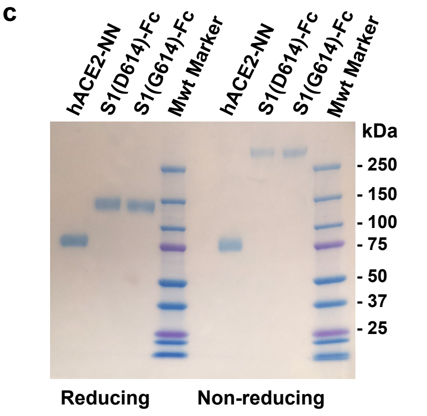

d

	S1(D614)-Fc x ACE2			S1(G614)-Fc x ACE2		
	k_{on} ($M^{-1}s^{-1}$)	k_{off} (s^{-1})	K_D (M)	k_{on} ($M^{-1}s^{-1}$)	k_{off} (s^{-1})	K_D (M)
1st	8.35×10^4	8.56×10^{-3}	1.03×10^{-7}	7.92×10^4	8.51×10^{-3}	1.07×10^{-7}
2nd	8.46×10^4	8.35×10^{-3}	0.99×10^{-7}	8.12×10^4	8.27×10^{-3}	1.02×10^{-7}
3rd	8.49×10^4	8.37×10^{-3}	0.99×10^{-7}	8.26×10^4	8.33×10^{-3}	1.01×10^{-7}

Supplementary Figure 9. Affinity of S^{G614} for hACE2 is not increased. The same data presented in Fig. 4a,b before normalized to total S protein level. **a,** The S protein containing C-terminal FLAG tag is transfected into HEK293T cells and assessed for hACE2-NN-Ig binding. Total S protein was measured by detecting the FLAG tag in the permeabilized cells. Anti-hIgG-FITC is used to measure ACE2-Ig binding and anti-mIgG-APC to measure FLAG level. **b,** Experiments similar to those in (a) except the S protein contains N-Myc and C-FLAG tags. The S1 level was assessed using an anti-Myc antibody together with anti-mIgG-APC and total S protein level measured by anti-FLAG followed by anti-mIgG-APC. Each symbol in (a,b) indicates an average value of a duplicated experiment. Mean ± SEM of n=3 independent experiments are presented. **c,** The proteins used in the SPR experiments shown in Fig. 4c were visualized by Coomassie-blue stain following SDS-PAGE. **d,** Association (k_{on}) and dissociation (k_{off}) rate constants and the equilibrium dissociation constant (K_D) obtained from three independent biacore assays are shown. Sensograms are shown in Fig. 4c. M.F.I.: mean fluorescence intensity.

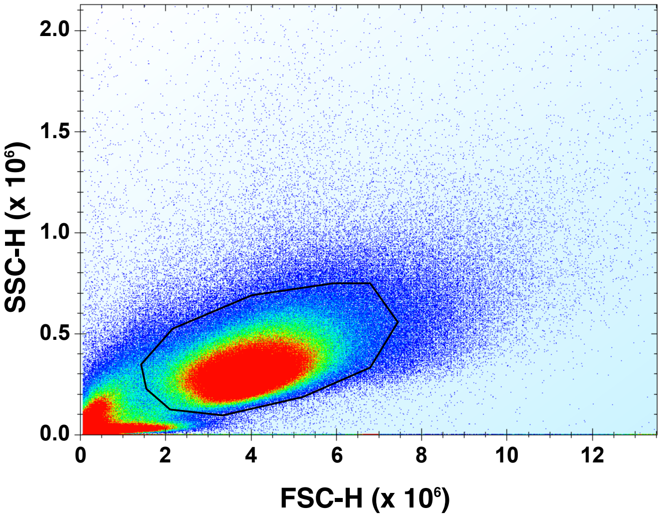

Supplementary Figure 10. Cell gating strategy for flow cytometry. For single-color flow cytometry experiments, gating on forward scatter (FSC) and side scatter (SSC) of the acquired events was performed to exclude debris and dead cells. The region of interest is defined by the black polygon. This approach was applied to measure GFP expression in Figures 1c, 1d, 1e, 1f, and 2b, or to measure antigen staining in Figures 4a, 4b, and Supplementary Figures 1c, 3b, 3c, 4b, 9a, and 9b.
