# Supplementary material for: SARS-CoV-2 spike-protein D614G mutation increases virion spike density and infectivity
Source: Nat Commun. 2020 Nov 26;11:6013. doi: 10.1038/s41467-020-19808-4 (PMC7693302; doi:10.1038/s41467-020-19808-4)
Supplement: Supplementary file 3 — Reporting Summary [file 41467_2020_19808_MOESM3_ESM.pdf]

nature research

Corresponding author(s): Michael Farzan and Hyeryun Choe

Last updated by author(s): Oct 7, 2020

Reporting Summary

Nature Research wishes to improve the reproducibility of the work that we publish. This form provides structure for consistency and transparency in reporting. For further information on Nature Research policies, see our Editorial Policies and the Editorial Policy Checklist.

Statistics

For all statistical analyses, confirm that the following items are present in the figure legend, table legend, main text, or Methods section.

n/a	Confirmed	
☐	☒	The exact sample size (*n*) for each experimental group/condition, given as a discrete number and unit of measurement
☐	☒	A statement on whether measurements were taken from distinct samples or whether the same sample was measured repeatedly
☐	☒	The statistical test(s) used AND whether they are one- or two-sided<br>*Only common tests should be described solely by name; describe more complex techniques in the Methods section.*
☒	☐	A description of all covariates tested
☐	☒	A description of any assumptions or corrections, such as tests of normality and adjustment for multiple comparisons
☐	☒	A full description of the statistical parameters including central tendency (e.g. means) or other basic estimates (e.g. regression coefficient) AND variation (e.g. standard deviation) or associated estimates of uncertainty (e.g. confidence intervals)
☐	☒	For null hypothesis testing, the test statistic (e.g. *F*, *t*, *r*) with confidence intervals, effect sizes, degrees of freedom and *P* value noted<br>*Give P values as exact values whenever suitable.*
☒	☐	For Bayesian analysis, information on the choice of priors and Markov chain Monte Carlo settings
☒	☐	For hierarchical and complex designs, identification of the appropriate level for tests and full reporting of outcomes
☒	☐	Estimates of effect sizes (e.g. Cohen's *d*, Pearson's *r*), indicating how they were calculated

Our web collection on statistics for biologists contains articles on many of the points above.

Software and code

Policy information about availability of computer code

Data collection	Flow cytometry data were collected with Accuri C6 software 1.0.264 (BD) and ForeCyt 6.2R3 (IntelliCyt). Bioluminescence assay data were collected with SoftMax Pro 6.3 (Molecular Devices). Western blot data were collected with Image Lab 6.1 (Bio-Rad). Quantitative PCR data were collected with CFX Manager 3.1 (Bio-Rad). Surface plasmon resonance assay data were collected using Biacore X100 Control Software 2.0.1 (Cytiva) and Biacore X100 Evaluation Software version 2.0.1 (Cytiva) to analyze data. Logo plots were generated using WebLogo version 3.
Data analysis	Experimental data were analyzed with GraphPad Prism version 7 (GraphPad Software Inc). Sequence data were analyzed with R version 3.6.0 (R Foundation for Statistical Computing) and the Biostrings package version 2.52.

For manuscripts utilizing custom algorithms or software that are central to the research but not yet described in published literature, software must be made available to editors and reviewers. We strongly encourage code deposition in a community repository (e.g. GitHub). See the Nature Research guidelines for submitting code & software for further information.

Data

Policy information about availability of data

All manuscripts must include a data availability statement. This statement should provide the following information, where applicable:

- Accession codes, unique identifiers, or web links for publicly available datasets
- A list of figures that have associated raw data
- A description of any restrictions on data availability

Source Data are provided with this paper. The data used to generate the Cryo-EM images in Figure 1a are available from PBD entry 6VXX (http://www.rcsb.org/structure/6vxx). Sequences used to generate logo plots in Figure 1b are available from GenBank (https://www.ncbi.nlm.nih.gov/protein) by searching for "SARS-

CoV-2 spike" and filtering the result by the length of the protein (1272-1273). To separate the result by the month, retrieved sequences can be further filtered by the release date.

Field-specific reporting

Please select the one below that is the best fit for your research. If you are not sure, read the appropriate sections before making your selection.

☒ Life sciences ☐ Behavioural & social sciences ☐ Ecological, evolutionary & environmental sciences

For a reference copy of the document with all sections, see nature.com/documents/nr-reporting-summary-flat.pdf

Life sciences study design

All studies must disclose on these points even when the disclosure is negative.

Sample size	The sample size of three convalescent plasma donors was used because that was the maximum number of samples available to us. No sample size calculation was performed for other experiments because no other experiments involve population sampling. However, all experiments, except Western blots, were performed with at least two technical replicates in each experiment to ensure accuracy of the data. All experiments, including Western blots, were replicated in at least 3 independent experiments conducted with at least two independently prepared samples.
Data exclusions	No data were excluded from analyses.
Replication	All experiments were replicated in at least 3 independent experiments performed on different days with at least two independently prepared samples such as PVs or VLPs. Number of independent experiments is noted in the figure legends.
Randomization	Randomization does not apply to this study because there were no samples allocated into control and experimental groups. For example, each prep of S-D614, S-G614, and S-FKO pseudoviruses or virus-like particles were produced in parallel in each batch and used in experiments side by side, controlling one another. For human plasma samples in Fig. 4, each sample was tested in all conditions.
Blinding	Blinding was not performed because this is not a case-control study. The authors could not be blinded during assay performance because these experiments are in vitro studies that we performed with materials (e.g. psueodoviruses, cells) that we propagated or prepared ourselves. Further, blinding is not necessary because this study is exclusively based on quantitative measurement from in vitro experiments.

Reporting for specific materials, systems and methods

We require information from authors about some types of materials, experimental systems and methods used in many studies. Here, indicate whether each material, system or method listed is relevant to your study. If you are not sure if a list item applies to your research, read the appropriate section before selecting a response.

Materials & experimental systems

n/a	Involved in the study
☐	☒ Antibodies
☐	☒ Eukaryotic cell lines
☒	☐ Palaeontology and archaeology
☒	☐ Animals and other organisms
☒	☐ Human research participants
☒	☐ Clinical data
☒	☐ Dual use research of concern

Methods

n/a	Involved in the study
☒	☐ ChIP-seq
☐	☒ Flow cytometry
☒	☐ MRI-based neuroimaging

Antibodies

Antibodies used	Anti-Flag, clone M2, Sigma, Cat # F1804, Lot # SLBK1346V<br>Anti-Myc, clone 9E10, National Cell Culture Center (NCCC has changed its name to Cell Culture Company and this antibody is no longer available)<br>Anti-MLV-p30, clone 4B2, Abcam, ab130757, GR3260525-1, 4B2<br>anti-human ACE2, monoclonal, R&D Systems, Cat # MAB9332<br>anti-mouse-IgG(PE), polyclonal, Jackson ImmunoResearch, Cat # 115-116-146<br>anti-human IgG(FITC), polyclonal, Jackson ImmunoResearch, Cat # 109-096-170<br>anti-mouse-IgG(APC), polyclonal, Jackson ImmunoResearch, Cat # 115-136-071<br>anti-mouse IgG(HRP), polyclonal, Jackson ImmunoResearch, Cat # 115-036-062<br>anti-human IgG(poly-HRP), clone M907122/M98245, Fitzgerald, Cat # 61R-I166AHRP40, Lot # 9424<br>anti-hIgG, CH2 in Human antibody capture kit, Cytiva, Cat # BR-1008-39
Validation	Anti-Flag, clone M2, Sigma (Cat # F1804) specific recognize DYKDDDDK peptide and validated by the manufacturer and many laboratories for FC, ICC, WB, and IP

Anti-Myc, clone 9E10, was obtained from National Cell Culture Center but is not available any longer. Its specificity and applications were validated in the lab for FC, ICC, IP, and WB using various tagged and untagged proteins (Li et al., 2005, EMBO J; Radoshitzky et al., 2007, Nature; Huang et al., 2011, PLoS Pathog; Jemielity et al,, 2013, PLoS Pathog).
Anti-p30, clone 4B2, Abcam (ab130757) specifically recognizes MLV gag protein, p30, and was validated by the manufacturer for WB using recombinant p30.
Anti-hACE2, R&D Systems (Cat # MAB9332) recognizes human ACE2 in FC, and validated by the manufacturer for FC and IHC using hACE2-transfected HEK293T cells.

Eukaryotic cell lines

Policy information about cell lines

Cell line source(s)	HEK293T (Cat # CRL-3216) and Calu-3 (Cat # HTB-55) were purchased from ATCC. NCI-H1299 (ATCC CRL-5803D) and NCI-H1975 (ATCC CRL-5908) lung epithelial cell lines were obtained from Joseph Kissil (Scripps Research, FL).
Authentication	HEK293T and Calu-3 were not separately authenticated because they were purchased from ATCC with certificates. NCI-H1299 (FTA Barcode #: STRB3886) and NCI-H1975 (STRB3884) were recently authenticated by ATCC Cell Line Authentication Service, using Short Tandem Repeat (STR) analysis as described in 2012 in ANS Standard (ASN-0002) Authentication of Human Cell Lines: Standardization of STR Profiling by the ATCC Standards Development Organization (SDO) and in Capes-Davis et al., Match criteria for human cell line authentication: Where do we draw the line? Int. J. Cancer. 2012 Nov 8. doi: 10.1002/ijc.27931
Mycoplasma contamination	Cells were tested for the presence of mycoplasma before frozen, using two different methods: DAPI staining for the presence of mycoplasma DNA and PlasmoTest (InVivogen) that detects all mycoplasmas through TLR2-mediated recognition. Thawed cells were not tested for mycoplasma contamination, because they were tested negative before frozen, but maintained in the presence of prophylactic Plasmocin antimycotic (InVivoGen) at 2.5 mg/ml. Antimycotic is used only for maintenance, and cells used in experiments are plated in media lacking antimycotic.
Commonly misidentified lines (See ICLAC register)	No commonly misidentified cell lines were used.

Flow Cytometry

Plots

Confirm that:

☒ The axis labels state the marker and fluorochrome used (e.g. CD4-FITC).

☒ The axis scales are clearly visible. Include numbers along axes only for bottom left plot of group (a 'group' is an analysis of identical markers).

☒ All plots are contour plots with outliers or pseudocolor plots.

☒ A numerical value for number of cells or percentage (with statistics) is provided.

Methodology

Sample preparation	For staining, HEK293T and transfected HEK293T cells were detached and washed in PBS. NCI-H1299, NCI-H1975, and Calu-3 cells were detached by scraping in PBS containing 5 mM EDTA. For entry assays, cells were harvested by trypsinization.
Instrument	Data were acquired on an Accuri C6 flow cytometer (BD) with HyperCyt high-throughput sampler (IntelliCyt).
Software	Data were collected and analyzed with Accuri C6 software 1.0.264 (BD) and ForeCyt 6.2R3 (IntelliCyt).
Cell population abundance	Flow cytometry was used in this study to quantify fluorescence intensity of GFP reporter expressed from PV-infected cells, or to stain tagged S protein expression on transfected HEK293T cells, or to assess ACE2 expression on lung epithelial cells. Thus we analyzed the viable cell population, which was the majority of the whole population, gated in pseudocolor plots.
Gating strategy	No special gating was utilized except to exclude dead cells and debris. The major population corresponding to HEK293T single cell suspension roughly centered at FSC: 4 x 10^6 / SSC: 3 x 10^5.

☒ Tick this box to confirm that a figure exemplifying the gating strategy is provided in the Supplementary Information.
