# Supplementary material for: SARS-CoV-2 spike-protein D614G mutation increases virion spike density and infectivity
Source: Nat Commun. 2020 Nov 26;11:6013. doi: 10.1038/s41467-020-19808-4 (PMC7693302; doi:10.1038/s41467-020-19808-4)
Supplement: Supplementary file 2 — Peer Review File [file 41467_2020_19808_MOESM2_ESM.pdf]

Editorial Note: This manuscript has been previously reviewed at another journal that is not operating a transparent peer review scheme. The manuscript was considered suitable for publication without further review at Nature Communications.
